# Progress Toward Poliovirus Containment Implementation — Worldwide, 2018–2019

**DOI:** 10.15585/mmwr.mm6838a3

**Published:** 2019-09-27

**Authors:** Daphne B. Moffett, Anna Llewellyn, Harpal Singh, Eugene Saxentoff, Jeffrey Partridge, Maria Iakovenko, Sigrun Roesel, Humayun Asghar, Najam Baig, Varja Grabovac, Santosh Gurung, Nicksy Gumede-Moeletsi, Jacob Barnor, Andros Theo, Gloria Rey-Benito, Andrea Villalobos, Liliane Boualam, Joseph Swan, Roland W. Sutter, Ekkehart Pandel, Steven Wassilak, M. Steven Oberste, Ian Lewis, Michel Zaffran, MEng

**Affiliations:** ^1^World Health Organization, Geneva, Switzerland; ^2^CDC; ^3^Bill and Melinda Gates Foundation, Seattle, Washington; ^4^Rotary International, Evanston, Illinois; ^5^United Nations Children's Fund, Geneva, Switzerland; ^6^Global Polio Eradication Initiative Containment Management Group, Geneva, Switzerland.

Among the three wild poliovirus (WPV) types, type 2 (WPV2) was declared eradicated globally by the Global Commission for the Certification of Poliomyelitis Eradication (GCC) in 2015. Subsequently, in 2016, a global withdrawal of Sabin type 2 oral poliovirus vaccine (OPV2) from routine use, through a synchronized switch from the trivalent formulation of oral poliovirus vaccine (tOPV, containing vaccine virus types 1, 2, and 3) to the bivalent form (bOPV, containing types 1 and 3), was implemented. WPV type 3 (WPV3), last detected in 2012 (*1*), will possibly be declared eradicated in late 2019.* To ensure that polioviruses are not reintroduced to the human population after eradication, World Health Organization (WHO) Member States committed in 2015 to containing all polioviruses in poliovirus-essential facilities (PEFs) that are certified to meet stringent containment criteria; implementation of containment activities began that year for facilities retaining type 2 polioviruses (PV2), including type 2 oral poliovirus vaccine (OPV) materials (*2*). As of August 1, 2019, 26 countries have nominated 74 PEFs to retain PV2 materials. Twenty-five of these countries have established national authorities for containment (NACs), which are institutions nominated by ministries of health or equivalent bodies to be responsible for poliovirus containment certification. All designated PEFs are required to be enrolled in the certification process by December 31, 2019 (*3*). When GCC certifies WPV3 eradication, WPV3 and vaccine-derived poliovirus (VDPV) type 3 materials will also be required to be contained, leading to a temporary increase in the number of designated PEFs. When safer alternatives to wild and OPV/Sabin strains that do not require containment conditions are available for diagnostic and serologic testing, the number of PEFs will decrease. Facilities continuing to work with polioviruses after global eradication must minimize the risk for reintroduction into communities by adopting effective biorisk management practices.

## Background

Since the Global Polio Eradication Initiative began, the number of reported WPV cases has declined from an estimated 350,000 WPV cases in 125 countries during 1988 to 66 cases in two countries with ongoing endemic transmission during 2019 (as of August 20, 2019); an estimated 18 million paralytic poliomyelitis cases have been prevented during the past 30 years.^†^ Although WPV transmission is now limited to two countries, 14 countries (as of September 17, 2019) currently have circulating VDPVs (cVDPVs) (i.e., rare strains of poliovirus that have genetically mutated from the vaccine strain and reverted to neurovirulence during replication as they circulate in communities) (Global Polio Eradication Initiative, unpublished data, 2019). cVDPVs can emerge in areas with low immunization coverage and cause outbreaks of paralytic poliomyelitis. Immunodeficiency-associated VDPVs can emerge in persons with primary immunodeficiencies and can be excreted for years, even by persons who are treated for their immunodeficiency. Immunodeficiency-associated VDPVs are rare; 111 cases have been documented since 1962. To provide immunity to type 2 poliovirus, a single dose of inactivated poliovirus vaccine (IPV) was introduced into the immunization schedule in most OPV-using countries before the global switch from tOPV to bOPV in 2016, and more recently in all other OPV-using countries. IPV provides serologic immunity to all three types of poliovirus, resulting in protection against paralytic poliomyelitis. However, studies indicate that the extent of mucosal immunity in the intestine conferred by IPV is significantly less than that provided by OPV (*4*); therefore, OPV continues to be used for outbreak responses to stop poliovirus transmission. When WPV eradication is achieved, countries hosting PEFs should continue the use of IPV, and all other countries without PEFs should maintain IPV in their routine immunization schedule for at least 10 years after global withdrawal of all OPV (*5*).

Once global polio eradication is achieved, and mass vaccination campaigns are no longer conducted, population immunity to polioviruses will decline. Thus, the consequences of any poliovirus introduction into communities from a facility containment breach would be severe. To mitigate this risk, all 194 WHO Member States resolved at the 68th World Health Assembly in 2015 to ensure that all polioviruses would be held only in specially certified poliovirus containment facilities (*6*). A revised WHO Global Action Plan to minimize poliovirus facility-associated risk after type-specific eradication of wild polioviruses and sequential cessation of oral polio vaccine use (GAPIII), released in 2015 (*5*), outlines the biorisk management requirements for laboratories, vaccine production sites, and other facilities retaining polioviruses (Figure). The Containment Certification Scheme to support GAPIII (GAPIII-CCS), which defined a process for independent certification of facilities, was endorsed by the WHO Strategic Advisory Group of Experts on Immunization (SAGE) and released in 2016 (*7*).

**FIGURE:**
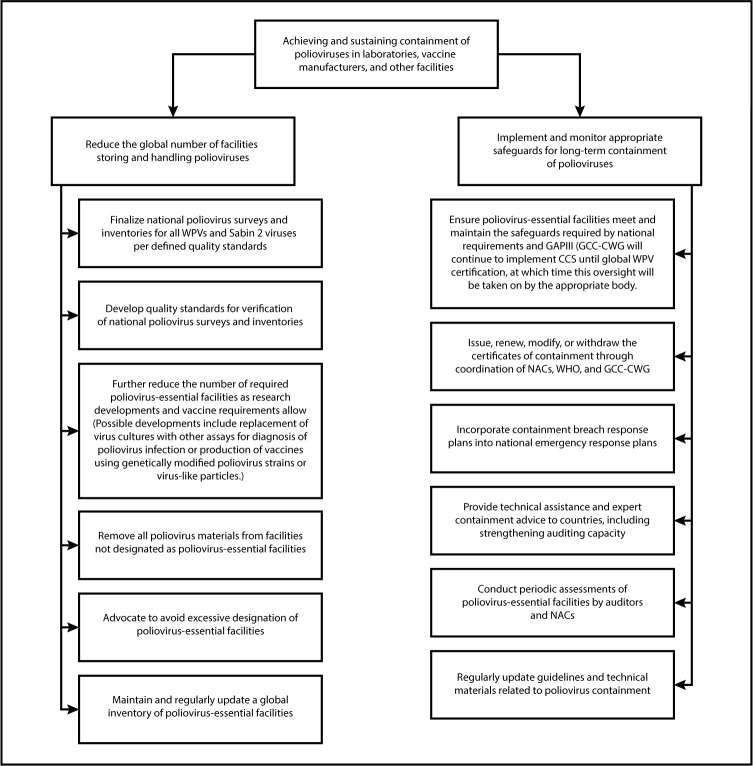
Planned major activities of the Polio Endgame Strategy — worldwide, 2019–2023 **Abbreviations:** CCS = Containment Certification Scheme; GAPIII = Third Global Action Plan to minimize poliovirus facility–associated risk after type-specific eradication of wild polioviruses and sequential cessation of oral poliovirus vaccine use; GCC-CWG = Global Commission for the Certification of Poliomyelitis Eradication Containment Working Group; NAC = national authority for containment; PEF = poliovirus-essential facility; WHO = World Health Organization; WPV = wild poliovirus.

## Global Poliovirus Containment Progress

GAPIII outlined a phased approach to poliovirus containment, beginning with PV2 materials. Phase I focuses on national facility surveys to identify and reduce the number of facilities retaining those materials; Phase II outlines activities related to certification of all PEFs retaining PV2 materials; and Phase III includes the final containment of all types of WPV, VDPV, and OPV/Sabin polioviruses (*2*). Phase I and Phase II activities are currently being implemented in parallel. A previous report indicated that Phase I inventories for facilities retaining PV2 infectious materials were complete (*8*). However, the most recent data show that because of previous underreporting, some WHO regions report an increase in the number of facilities holding PV2 materials, although the number of PEFs has decreased globally. In 2016, approximately 100 PV2 facilities worldwide intended to become GAPIII-certified; this number declined to 86 in 2017, to 81 in 2018, and to 74 as of August 1, 2019 (Table). This reduction in candidate PEFs occurred in part because many facility managers elected not to implement the rigorous requirements for containment certification and have ceased or will soon cease working with PV2 materials. The remaining 74 facilities are located in 26 countries, 25 of which have established NACs to oversee facility compliance and certification, with the final country working through its domestic legal process to establish a NAC.

**TABLE:** Number of designated poliovirus-essential facilities (PEFs) retaining poliovirus type 2 (PV2) materials* and established national authorities for containment (NACs), by World Health Organization (WHO) region — worldwide, August 2019^†^

WHO region	No. of countries	No. of NACs	No. of PEFs	Type of PV2 materials retained, no. of facilities			Facility types, no.		
				Only WPV/VDPV type 2	Both WPV/VDPV type 2 and OPV/Sabin type 2	Only OPV/Sabin type 2	Vaccine production		Diagnostic or research laboratories
							Salk (WPV)-IPV	Sabin-IPV^§^	
African Region	1	1	1	0	1	0	0	0	1
Region of the Americas	5	5	18	6	4	8	1	0	17
Eastern Mediterranean Region^¶^	2	2	3	0	1	1	0	2	1
European Region	11	10	34	3	20	11	5	2	27
South-East Asia Region	2	2	2	0	1	1	0	1	1
Western Pacific Region	5	5	16	0	4	12	0	11	5
**Total**	**26**	**25**	**74**	**9**	**31**	**33**	**6**	**16**	**52**

**Abbreviations**: IPV = inactivated poliovirus vaccine; OPV = oral poliovirus vaccine; VDPV2 = type 2 vaccine-derived poliovirus; WPV = wild poliovirus; WPV2 = wild poliovirus type 2.

* Includes WPV2/VDPV2 and OPV2/Sabin type 2.

^†^ Data as of August 1, 2019.

^§^ Includes potential future producers in different clinical and preclinical phases of Sabin-IPV development.

^¶^ One PEF in this region does not currently retain PV2 material but is pursuing certification for future work.

In addition to identifying facilities retaining WPV and cVDPVs, countries are also required to identify laboratories retaining potentially infectious materials (i.e., specimens collected for other purposes in countries where WPV and cVDPVs were in circulation). Laboratories with a high probability of handling or storing potentially infectious poliovirus materials include those working with enteric or respiratory disease agents and facilities engaged in nutrition research or environmental studies. To aid countries in identifying facilities retaining potentially infectious materials, WHO published Guidance to Minimize Risks for Facilities Collecting, Handling or Storing Materials Potentially Infectious for Polioviruses in 2018 (*9*). The rollout of this guidance included ongoing country technical support, targeted country visits, webinars, and WHO-led national and regional workshops. Global implementation of this guidance has been challenging because of its labor-intensive nature and application to thousands of laboratories worldwide.

By resolution of WHO Member States at the 71st World Health Assembly in 2018, all facilities designated to retain PV2 materials (including OPV2/Sabin type 2 infectious materials) are required to be enrolled in the certification process through NACs by December 31, 2019. To date, seven NACs have submitted 13 applications to GCC; seven have been accepted by the GCC Containment Working Group, which conducts the reviews.^§^ Facility auditing by GAPIII-CCS-qualified auditors is required to certify a PEF. Auditor qualification is ongoing; 142 auditor trainees from 27 countries have passed preliminary GAPIII-CCS training, and 10 lead auditors will be fully qualified by the end of 2020.

If WPV3 is declared eradicated, poliovirus type 3 containment would begin with a focus on WPV3 and VDPV type 3. OPV3/Sabin type 3 containment processes would not begin until OPV3 is withdrawn from routine immunization programs and campaigns, currently scheduled as part of the global withdrawal of bOPV after WPV type 1 (WPV1) is also eradicated. The 71st World Health Assembly resolution urged countries to accelerate completion of national surveys for WPV1 and WPV3 infectious and potentially infectious materials (*10*).

## cVDPV2 Outbreak Containment Challenges

After declaration of WPV2 eradication in 2015, a coordinated global switch from tOPV to bOPV for routine immunization and supplementary immunization activities took place in 2016. To mitigate the risks associated with the withdrawal of OPV2, SAGE recommended that all OPV-using countries introduce at least 1 dose of IPV into their routine immunization program. As a result of challenges in reaching unimmunized and underimmunized children in some areas before the switch, an increasing number of circulating VDPV type 2 (cVDPV2) outbreaks have been reported since the switch, including three in 2016, four in 2017, six in 2018, and 14 to date in 2019. The increasing number of cVDPV2 outbreaks after the switch has led to a corresponding increase in monovalent OPV2 (mOPV2, containing type 2 vaccine virus) outbreak response immunization activities, resulting in a projected administration of 312 million doses by the end of 2019. The ongoing challenges with cVDPV2 outbreaks and the increased need for mOPV2 could lead vaccine manufacturers to restart mOPV2 production and enrolling facilities in GAPIII certification, increasing the global poliovirus containment workload. Intensified coordination among multiple WHO and United Nations Children's Fund teams and country authorities will be crucial to ensuring uninterrupted availability of mOPV2 produced under applicable biorisk management controls. cVDPV2 outbreaks subsequent to mOPV2 use will require countries with facilities handling infectious and potentially infectious materials to repeat PV2 surveys once the outbreaks have ended.

## Discussion

The new Global Polio Eradication Initiative Polio Endgame Strategy 2019–2023 (*1*) contains three important pillars: eradication, integration, and containment/certification. The containment section focuses on further reducing the number of PEFs and the implementation and monitoring of safeguards for long-term containment of polioviruses. After global eradication of all WPVs and eventual bOPV cessation, fully certified containment of all polioviruses in research and quality control laboratories, vaccine manufacturing facilities, biomedical facilities, and biological repositories is crucial. Containment efforts include minimizing the number of facilities retaining poliovirus materials and ensuring that all poliovirus research facilities comply with containment guidelines. Ongoing poliovirus research facilitates the development and deployment of alternative, genetically stable polioviruses that are safe to use in vaccination and that can be produced and used outside containment.

Researchers have made important progress in replacing Sabin strains for diagnostic and serologic assays (e.g., with genetically stable novel OPVs) (*4*) and in developing IPVs made from Sabin and safer poliovirus strains to reduce risks from the use of live WPV in IPV production. These advances will result in a requirement for fewer poliovirus containment facilities and a corresponding reduction in overall risk for poliovirus release.

Since GAPIII was published in 2015, the addition of Guidance to Minimize Risks for Facilities Collecting, Handling or Storing Materials Potentially Infectious for Polioviruses and recommendations from the WHO Containment Advisory Group have resulted in modifications to poliovirus containment requirements, including the removal of full GAPIII requirements for handling poliovirus RNA and OPV2/Sabin type 2 potentially infectious material.^¶^^,^** An update to GAPIII, highlighting these approved changes, is anticipated by the end of 2020. After polio eradication, maintaining the global polio-free status will require vigilance and, for facilities retaining poliovirus, strict adherence to GAPIII requirements.

**Summary**

**What is already known about this topic?**

After certification of eradication of wild poliovirus type 2 in 2015, World Health Organization Member States committed to contain all poliovirus materials safely.

**What is added by this report?**

Twenty-six countries have designated 74 poliovirus type 2 poliovirus-essential facilities to retain poliovirus type 2 materials; these countries need to begin the certification process before the end of 2019. Upon certification of wild poliovirus type 3 eradication and expanded manufacture of monovalent oral poliovirus type 2 to combat ongoing vaccine-derived poliovirus type 2 outbreaks, the number of designated poliovirus-essential facilities will increase.

**What are the implications for public health practice?**

After the world is certified polio-free, all poliovirus serotypes ultimately will require secure containment because any release into communities could result in widespread transmission.

## Footnotes

* http://polioeradication.org/wp-content/uploads/2016/07/GCC-report-26-27-Feb-2019-20190227.pdf.

† https://www.who.int/news-room/fact-sheets/detail/poliomyelitis.

§ http://polioeradication.org/wp-content/uploads/2016/07/rolling-timeline-for-containment-certification-applications-20190710.pdf.

¶ http://polioeradication.org/polio-today/preparing-for-a-polio-free-world/containment/containment-supporting-groups/.

** http://polioeradication.org/tools-and-library/policy-reports/advisory-reports/containment-advisory-group/.
